# The Changes of Mitochondria in Substantia Nigra and Anterior Cerebral Cortex of Hepatic Encephalopathy Induced by Thioacetamide

**DOI:** 10.1002/ar.23932

**Published:** 2019-03-04

**Authors:** Yunhu Bai, Shengming Wang, Feifei Wu, Xiangjun Xie, Yayun Wang, Yanling Yang

**Affiliations:** ^1^ Department of Hepatobiliary Surgery Xi‐Jing Hospital, Fourth Military Medical University Xi'an China; ^2^ Department of Anatomy and K.K. Leung Brain Research Centre Fourth Military Medical University Xi'an China; ^3^ Department of Preventive Medicine The Fourth Military Medical University Xi'an China; ^4^ Department of general surgery People's Liberation Army's 153rd hospital Zhengzhou China

**Keywords:** mitochondria, substantia nigra, anterior cerebral cortex, hepatic encephalopathy, thioacetamide

## Abstract

Hepatic encephalopathy (HE) is a neuropsychiatric syndrome resulting from chronic or acute liver failure. Under the condition of HE, various factors such as reactive oxygen species, inflammatory factors, ammonia poisoning and amino acids alteration lead to changes of mitochondria. Selective depletion of damaged mitochondrion is essential for maintaining the morphology and function of mitochondria and cells. In this study, molecular biology analysis was used to analyze the mitochondrial morphology in the substantia nigra (SN) and anterior cerebral cortex (ACC) of the HE mice. The results revealed that the *drp1*, *mfn1* and *mfn2* increased in mRNA level of SN, which indicated the changes of mitochondrial morphology in HE mice. The *drp1* and *mfn2* genes were up‐regulated, then, the *Opa1* exhibited no significant change in the ACC of HE mice. Further study demonstrated that the mitochondrial autophagy related genes, *pink1* and *parkin,* increased in SN, while the *parkin* reduced in ACC of HE mice. In addition, uncoupling protein (*ucp2*) increased in mRNA level of SN and ACC, and the *ucp4* had no change or reduced in SN and ACC, respectively. These findings suggested that the mitochondrial dynamics is different in the SN and ACC of HE mice. Therefore, our results indicated that mitochondrial dynamics provided a potential treatment strategy for HE through the fission, fusion and autophagy of genes. Anat Rec, 302:1169–1177, 2019. © 2018 The Authors. *The Anatomical Record* published by Wiley Periodicals, Inc. on behalf of American Association of Anatomists.

Hepatic encephalopathy (HE) is a primary neurological disorder that occurs in patients with severe liver diseases (Williams, 1973). With acute HE, patients show the altered motor function and loss of coordination, including bradykinesia, psychomotor slowing, which seriously impact the quality of life. Acute HE has an extremely high mortality rate (80%–90%) due to the development of brain edema, increased intracranial pressure, metabolic disorders (decrease of ATP), inflammation, the accumulation of reactive oxygen and nitrogen species (ROS and RNS; Hindfelt and Siesjo, 1970; Hindfelt et al., 1977; Capocaccia and Angelico, 1991). Increasing of the brain and blood ammonia level was closely associated with the brain edema and disturbance energy metabolism in the HE mice. In the 1890s, Pavlov and Nencki hypothesized that ammonia involved in the pathogenesis of HE mice. In recent decades, studies suggested that ammonia poisoning, manganese poisoning, neurotransmitters played an important role in HE. However, the mechanisms of HE were still unclear. Now, increasing number of studies have focused on the relationship between mitochondria and HE (Bustamante et al., 2011; Rama and Norenberg, 2012).

The functions of mitochondria were inseparable from the production of ROS and ATP. Current researches indicated that the changes of cerebral energy metabolism presented in acute and chronic HE and hyperammonemia contained the altered glycolysis, tricarboxylic acid cycle and the electron transport chain (Panatto et al., 2011). Another possible mechanism for impaired energy metabolism and brain edema in HE and hyperammonemia might be the mitochondrial permeability transition (mPT; Reddy et al., 2009). Some researchers believed that ROS was one of the main causes of HE pathology (Skowronska and Albrecht, 2013). Oxidative stress not only induced mitochondrial fission but also interfered with mitochondrial regeneration. In addition, disturbances of mitochondrial dynamic and regenerative in turn further enhanced the level of ROS (Guo et al., 2015).

All of characteristics described above suggest that mitochondria related dynamic had pivotal influence on HE and hyperammonemia. Nevertheless, mitochondrial morphology was controlled by the different genes of fission and fusion, including mitochondrial fission 1 (FIS1), dynamin‐related protein 1 (DRP1) and mitochondrial fission factor, while fusion was regulated by optic atrophy (OPA1), mitofusin‐1 (MFN1) and mitofusin‐2 (MFN2; Rovira‐Llopis et al., 2017). PINK1 and PARKIN participated in the process of mitophagy, which was responsible for mitochondrial fission and fusion. Mitochondria dynamics had an important effect on cell protection after being stimulated (Yang et al., 2008; Wong and Holzbaur, 2015). The uncoupling proteins 2/4 (UCP2/4) belonged to the superfamily of mitochondrial carriers that were alleged to carry metabolic substrates (H^+^) across the mitochondrial inner membrane. It was generally accepted that the primary function of UCP2/4 regulated ROS, ATP and mitochondrial membrane potential (MMP; Hao, 2009; Ruiz‐Ramirez et al., 2011). Therefore, we hypothesized that mitochondria were disrupted in substantia nigra (SN) and anterior cerebral cortex (ACC) under the condition of HE, which could induce abnormal mitochondrial morphology and function.

In this study, we established animal model of acute HE on mice, which induced by intraperitoneal injection of thioacetamide (TAA) and investigated the change of mRNA level in mitochondrial dynamic related genes, including mitochondrial fusion, fission and autophagy in the SN and ACC of the HE mice.

## MATERIAL AND METHODS

### Animals

The adult male C57/BL mice (a total of 40) weighing 18–23 g were used for the experiments. Animals were divided into four groups randomly, including sham group, HE 1, 4, and 7 day groups (each group of 10). The animals were maintained in a temperature‐controlled environment under a 12‐hr light/dark cycle with free access to food and water. All the experimental steps on the animals were conducted in complied with Institutional Animal and The Fourth Military Medical University (China, Shaanxi) and the National Institutes of Health Guide for the care and use of laboratory animals.

### The Model of Acute HE

The HE model was constructed as previously reported (Miranda et al., 2010). Briefly, HE was induced by daily intraperitoneal (i.p.) injections of TAA (Sigma, USA) (300 mg/kg, dissolved in 0.3 mL 0.9% NaCl) for three consecutive days. The sham group received 0.3 mL of 0.9% NaCl (i.p.). In order to preventing electrolyte imbalance, hypoglycemia and renal failure, HE groups and sham group received supportive therapy consisting of 0.5 mL 0.45% NaCl, 5% glucose and 0.2% KCl every 12 hr after the first injection of TAA. After experimental treatments, maintained the normal body temperature of mice.

### Behavioral Assessments

A series of behavioral assessments were used to evaluate the behavioral phenotype of C57/BL mice as a model of neuropsychiatric illness. All behavioral testing were carried out in the same environment.

### Open Field Examinations Test

The open field examinations (OPE) test was carried out in polyurethane arena (50 × 50 × 45 cm^3^). Mice were put in center of the apparatus locomotion and recorded the center activity distance and total distance for 5 min. After each test, square box were disinfected and wiped out of odors of other animals with 75% ethanol. Finally, the ANY‐Maze Software was utilized to record and analyze data of behavior.

### Elevated Plus Maze Test

To assess the effect of stress on neuropsychiatric behavior, elevated plus maze (EPM) test was used. In brief, the EPM apparatus was made up of two open arm (40 × 10 cm^2^) and two closed arms (40 × 10 × 25 cm^3^) facing each other and connected by a central platform (10 × 10 cm^2^). The maze was elevated 40 cm above the floor. Testing began by placing each mouse in the central platform of the maze facing one of the open arms. An entry was registered only when all four paws of the animal were placed into an arm. Percentage of open arm access times and open arm retention time in each arm were manually recorded in the period of 5 min. After each test, all facilities were disinfected and wiped out of odors of other animals with 75% ethanol. Finally, the ANY‐Maze Software was employed to record and analyze data of behavior.

### Liver Function and Blood Ammonia Detection

Hepatic damage was evaluated by the levels of alanine aminotransferase (ALT) and aspartate aminotransferase (AST) in serum, according to standard reagent kits (Tiangen, Shanghai, China). Blood ammonia was detected in accordance with the commercial blood ammonia detection kit, following the manufacturer's protocol (mlbio, shanghai, China). In short, serum samples were collected by centrifugation (2,500 rpm/min, 15 min). First, adding the different concentrations of samples to kit that has been added the enzyme substrate in advance, and then add the color reagent. After cleaning for five times, absorbance was determined at 450 nm. Finally, standard curve was used to calculate the concentrations of ammonia in samples.

### Hematoxylin–Eosin Stain

The liver from each mouse was soaked in 10% formalin, embedded in paraffin, cut into 4 μm sections, and stained with Hematoxylin–Eosin (H&E) stain. We then observed the degree of injury in the liver tissue according to inflammatory cell infiltration and necrosis of hepatocytes. Finally, the photographs were taken under the light microscope (Olympus, Japan).

### Quantitative Real‐Time Polymerase Chain Reaction Analysis

Total RNA was extracted from the SN in all groups using Trizol (Invitrogen Life Technologies, Carlsbad, CA). Then, cDNA was created using the total RNA from the SN. Quantitative real‐time polymerase chain reaction (qRT‐PCR) was performed by SYBR green PCR master mix (Takara, Tokyo, Japan). Gene‐specific primers were as follows (See Table 1). Twenty‐five microliters of qRT‐PCR reaction systems were used, and the reaction conditions were as follows: 95°C denaturation for 5 sec, 60°C annealing extension for 30 sec, for a total of 39 cycles, the last plus dissolution curve. qRT‐PCR products were measured using a comparative ΔCT method and 2^−ΔΔCT^ to represent the relative expression of the target gene. All of the assays were corrected by subtracting 18S (Shenggong, China).

**Table 1:** Primers used for quantitative real‐time polymerase chain reaction

Gene name	Primer name	Primer sequence (5′→3′)
*ucp2*	F	TGG GAG GTA GCA GGA AAT CA
	R	GCG GTA TCC AGA GGG AAA GT
*ucp4*	F	CTC AGA GCC AAC CGA ATA GC
	R	GGC TGA CAG ATG CAA CAG AA
*mfn1*	F	GGT CTG CTT TCC TGC TCT CT
	R	CTT TCT GCT CCC ATT TCA CC
*mfn2*	F	CCT GGG ATC GAT GTT ACC AC
	R	AAC TGC TTC TCC GTC TGC AT
*drp1*	F	AAC AGG CAA CTG GAG AGG AA
	R	GCA ACT GGA ACT GGC ACA TC
*pink1*	F	CCT GGC TAC CAT GAT GAC CT
	R	ACA GCC ATC TGA GTC CCA CT
*parkin*	F	ATC TTG CTG GGA CGA TGT CT
	R	CCT TGT CTG AGG TT G GGT GT
*opa1*	F	GCC TTC CTC TTC GTC TCT CC
	R	CTC ACT TGC TTC CAC ACC AA

### Statistical Analysis

Statistical analysis was performed using SPSS 13.0 software and data were expression as the mean ± *SD*. The data were determined using one‐way analysis of variance (ANOVA). *P* value <0.05 was considered as statistically significant.

## RESULTS

### Liver Injury and Blood Ammonia Level

**Figure 1:**
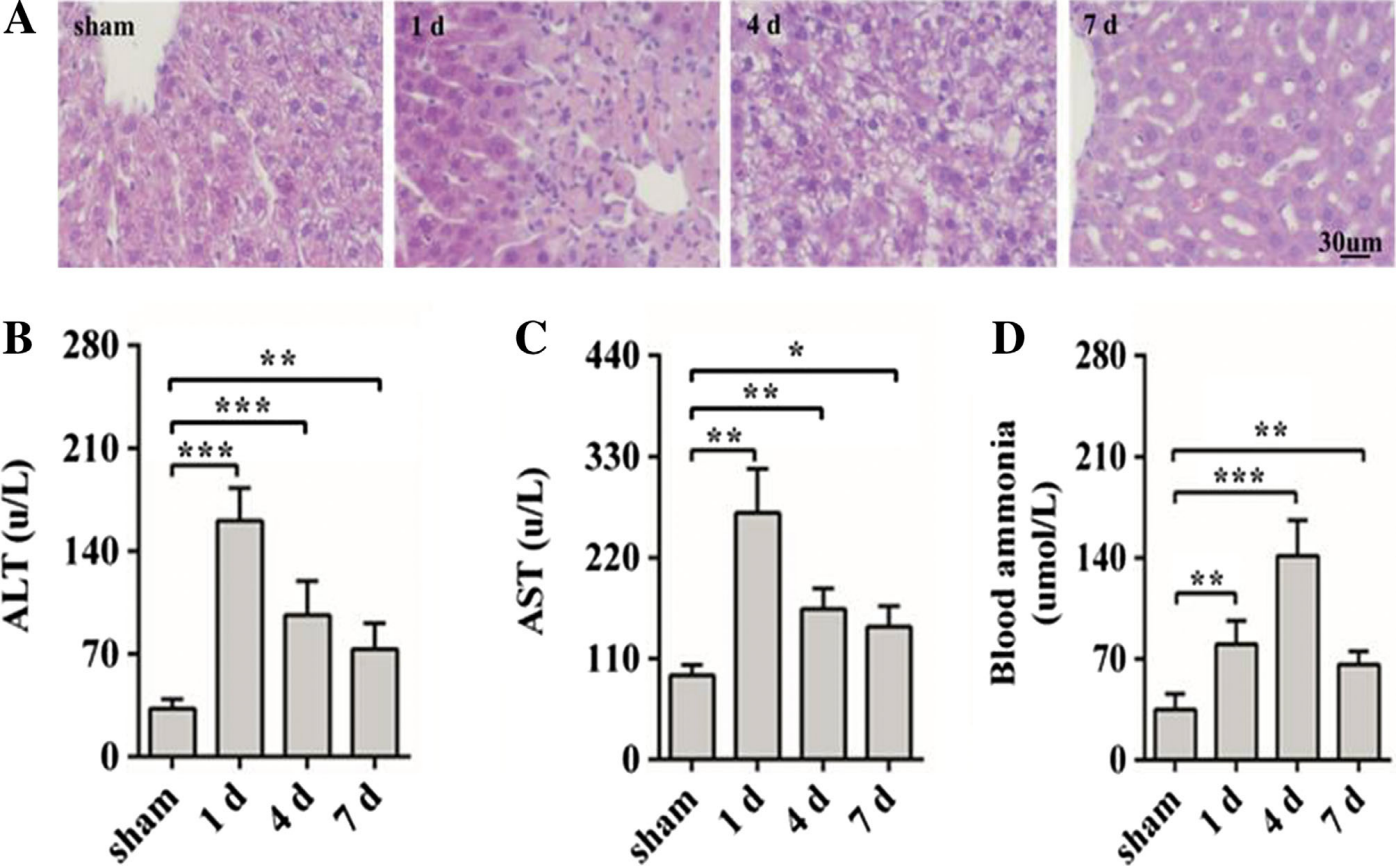
Liver function, pathology and blood ammonia. (**A**) Increased necrosis of hepatocytes observed in mice of HE groups (1 and 4 days). *n* = 4 per group. Scale bar = 50 μm. The level of ALT (**B**), AST (**C**) and blood ammonia (**D**) in sham and HE groups. ALT and AST significantly increased in HE groups compared with the sham group. Blood ammonia also increased in HE group. ^*^ *P* < 0.05, ^**^ *P* < 0.01, ^***^ *P* < 0.001, compared with the sham group. *n* = 8 per group. ALT, alanine aminotransferase; AST, aspartate aminotransferase; HE, hepatic encephalopathy.

To investigate whether injected the TAA induces injury of liver, we detected the ALT, AST, blood ammonia and hepatocyte with HE staining. The levels of ALT and AST significantly increased in the model of HE mice (Fig. 1B,C). Moreover, the concentration of blood ammonia markedly enhanced in HE 1 and 4 days compared with the sham group (Fig. 1D). In addition, H&E stain showed that a large number of hepatocytes in 1 and 4 day groups necrosis, then 7 day groups were more relieved (Fig. 1A). In details, H&E stain revealed that liver cells were in good shape and hepatic cords were evenly distributed in sham group. After 1 and 4 days of TAA injection, hepatocytes were significantly deformed and necrotic with a large number of inflammatory cells infiltrated. Until 7 days, the structure of hepatocytes recovered partially. The above results indicated that the groups of HE had obvious liver injury.

### Reduced the Locomotion and Mood in HE Mice

**Figure 2:**
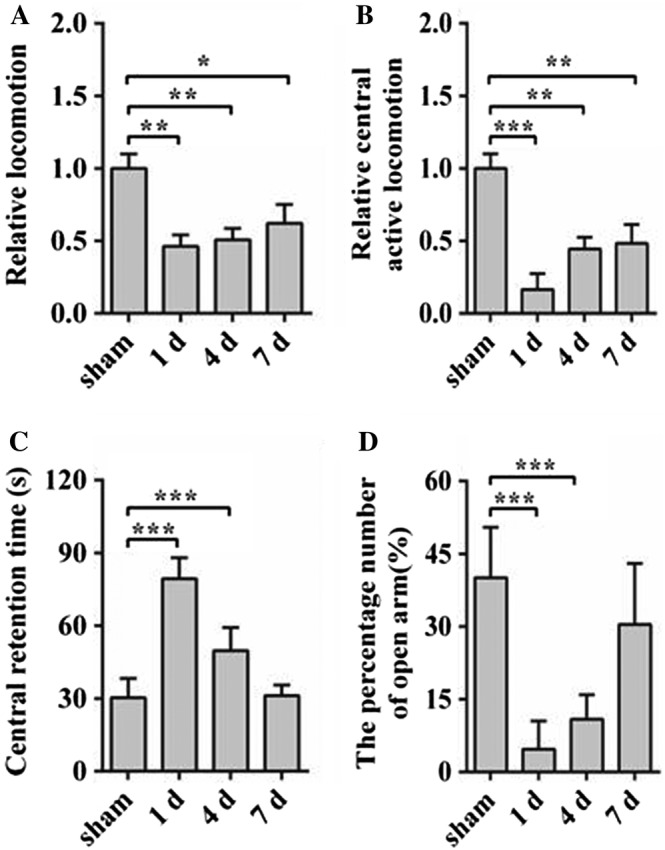
The changes of locomotion and mood induced by HE. (**A**) The open field experiment showing the reductions of total distance and central activity distance in the HE 1, 4, and 7 day group. (**B**) Elevated plus maze test revealing the decrease of central retention time and the percentage number of open arm in the HE 1 and 4 day group. Data are presented as mean ± *SD*. ^*^ *P* < 0.05, ^**^ *P* < 0.01, ^***^ *P* < 0.001, compared with the sham group. *n* = 10 per group. HE, hepatic encephalopathy.

In order to further verify whether the injection of TAA resulted in the changes in the nervous system, we observed locomotion and mood by the OPE and EPM test. As shown in Figure 2, OPE test showed that HE groups exhibited significantly reduced the total distance (Fig. 2A) and the center activity distance (Fig. 2B) in the OPE test compared with the sham group. In EPM test, HE groups also showed increased central retention time (Fig. 2C) and decreased enter the number of open arm (Fig. 2D) compared with the sham group. In short, these results revealed that HE could reduce locomotion and increase depression.

### The Change of the Fission and Fusion Genes in SN and ACC of HE Mice

**Figure 3:**
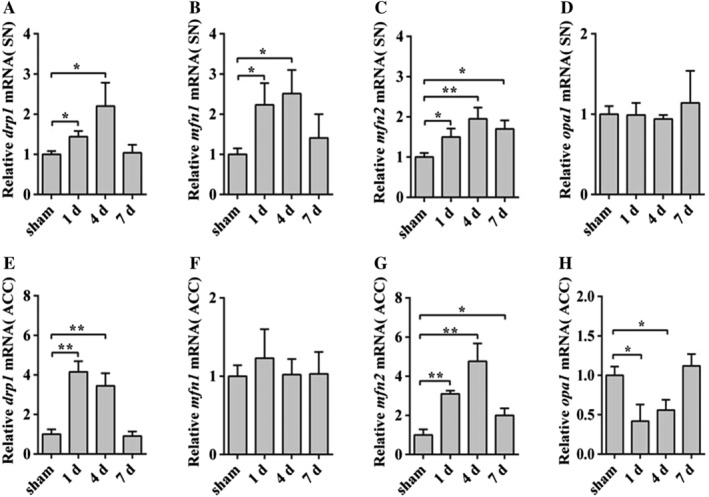
HE induces mitochondrial dynamic altering in SN and ACC of mice. qRT‐PCR analyses of *drp1* (**A** and **C**), *mfn1* (**B** and **F**), *mfn2* (**C** and **G**) and *opa1* (**D** and **H**) in SN and ACC of HE mice. Data are presented as mean ± *SD*. ^*^ *P* < 0.05, ^**^ *P* < 0.01, ^***^ *P* < 0.001, compared with the sham group. *n* = 3–4 per group. ACC, anterior cerebral cortex; HE, hepatic encephalopathy; qRT‐PCR, quantitative real‐time polymerase chain reaction; SN, substantia nigra.

To detect the changes in mitochondrial fragmentation, we measured the fission (*drp1*) and fusion (*mfn1/2*, *opa1*) genes of mitochondrial in SN and ACC in HE mice. The level of *drp1* mRNA significantly increased in the groups of 1 and 4 days in SN of HE compared with the sham group (Fig. 3A). And, we further investigated the expression levels of key regulators responsible for mitochondrial dynamic in SN and ACC of HE. The mRNA level of *mfn1/2* increased (Fig. 3B,C), then, the *opa1* did not alter in the level of mRNA in SN (Fig. 3D). The results indicated that the mitochondrial fission and fusion increased in SN of HE mice.

**Figure 4:**
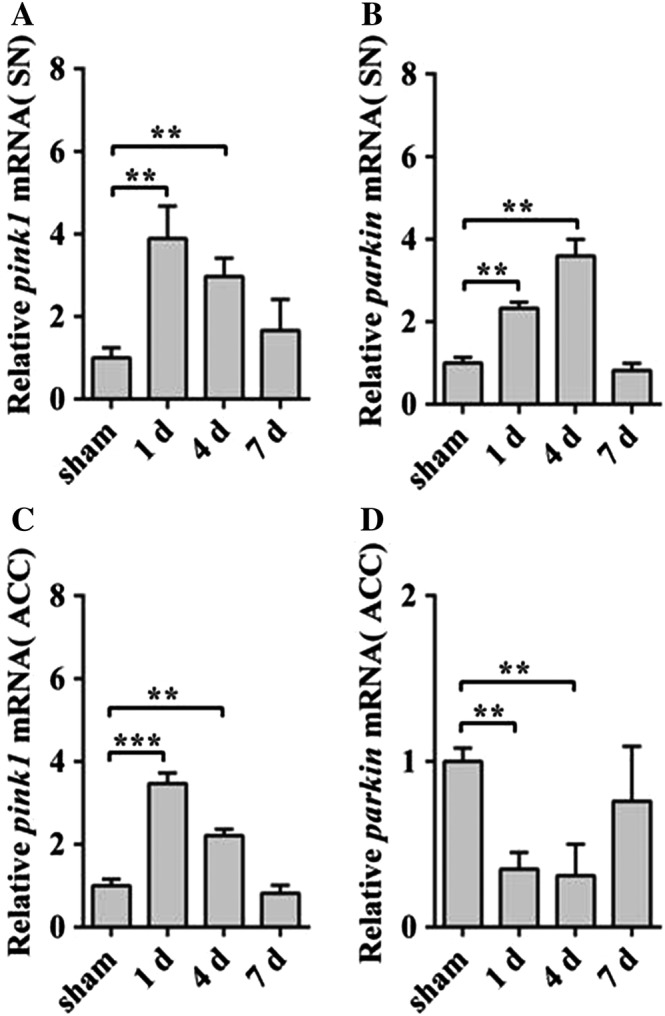
The autophagy increased in SN of HE mice. qRT‐PCR analyses of *pink1* (**A**) and *parkin* (**B**) in mRNA levels in SN of HE mice. qRT‐PCR analyses of *pink1* (**C**) and *parkin* (**D**) in ACC of HE groups. Data are presented as mean ± *SD*. ^*^ *P* < 0.05, ^**^ *P* < 0.01, ^***^ *P* < 0.001, compared with the sham group. *n* = 3–4 per group. ACC, anterior cerebral cortex; HE, hepatic encephalopathy; qRT‐PCR, quantitative real‐time polymerase chain reaction; SN, substantia nigra.

In ACC, the *drp1* increased in the HE groups of 1 and 4 days compared with the sham group (Fig. 4E). The level of *mfn2* increased (Fig. 4G) as well. However, *mfn1* and *opa1* had no change or reduced, respectively (Fig. 4F,H). These results showed that the mitochondrial fission increased and the fusion did not increase in the ACC.

### *Pink1* and *parkin* Promoted the Mitochondrial Autophagy in the SN, but Not in ACC of HE

To observation the autophagy of mitochondrion, we detected the genes *pink1*and *parkin* in the SN and ACC by qRT‐PCR analysis, which were associated with mitochondrial autophagy. The results showed that *pink1* and *parkin* significantly increased in the mRNA level in the SN of HE 1 and 4 days (Fig. 4A,B), while *pink1* increased (Fig. 4C) and *parkin* reduced (Fig. 4D) in ACC of HE mice, respectively. These results indicated that mitochondrial autophagy enhanced in the SN, but had no obvious change in ACC.

### Mitochondria Self‐Regulatory Function in HE

**Figure 5:**
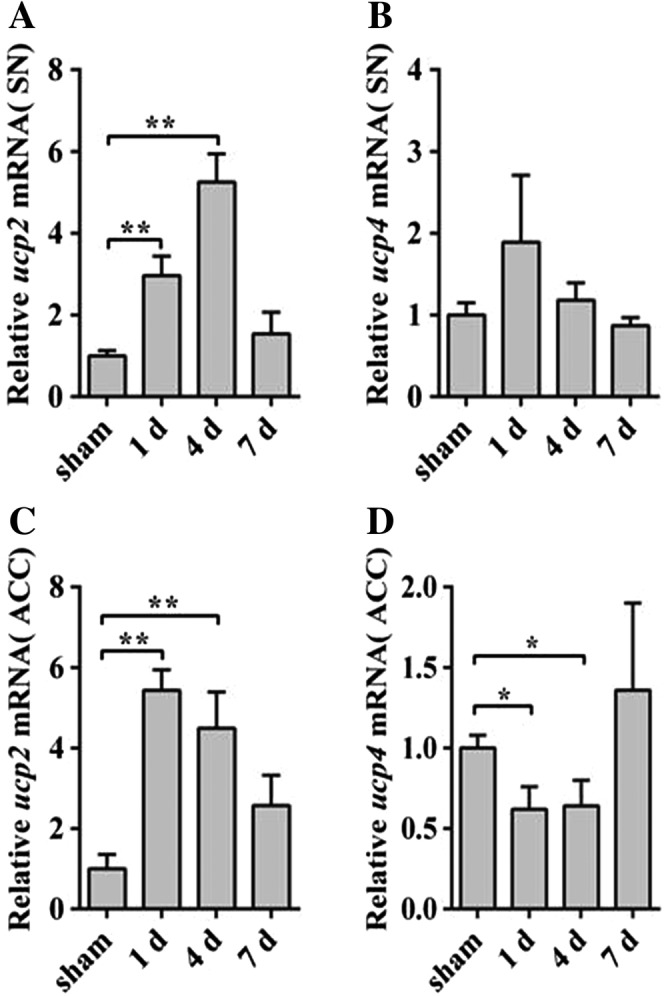
UCP2 increased in SN and ACC of HE mice. qRT‐PCR analyses of *ucp2* (**A**) and *ucp4* (**B**) in mRNA levels in SN of HE mice. Quantitative analyses of *ucp2* (**C**) and *ucp4* (**D**) by the qRT‐PCR in ACC of HE groups. Data are presented as mean ± *SD*. ^*^ *P* < 0.05, ^**^ *P* < 0.01, ^***^ *P* < 0.001, compared with the sham group. *n* = 3–4 per group. ACC, anterior cerebral cortex; HE, hepatic encephalopathy; qRT‐PCR, quantitative real‐time polymerase chain reaction; SN, substantia nigra.

In order to measure the function of mitochondria in the SN and ACC of HE, we detected the expression of the ucp2/4 in mRNA level of SN and ACC. UCP2/4 implicated in the regulation of body temperature, body weight, reduction of ROS, MMP, and ATP production. It was proposed that the UCP2 and UCP4 had important influences on cellular oxidative stress in the central nervous system (Andrews, 2005; Guevara et al., 2009). qRT‐PCR analysis found that *ucp2* increased in the SN and ACC of HE (Fig. 5A,C). The level of *ucp4* in mRNA did not change in SN of HE mice (Fig. 5B), while reduced in ACC (Fig. 5D).These results indicated that *ucp2* and *ucp4* had different effects on mitochondria in SN and ACC of HE mice**.**

## DISCUSSION

This study is the first to research alterations of mitochondrial dynamics and mitophagy in SN and ACC of HE mice induced by TAA. The mitochondrial dynamics and mitophagy regulated metabolism, cellular differentiation and neurodegeneration (Rovira‐Llopis et al., 2017). Mitochondria were fission and fusion constantly. The rapid modulation of mitochondrial dynamics occurs in response to physiological stimulation, including apoptotic stimulation (Spinazzi et al., 2008; Morciano et al., 2016), oxidative stress (Partyka et al., 2016; Rogov et al., 2018) to maintain the metabolic balance (Stockburger et al., 2016; Agnihotri et al., 2017). Therefore, mitochondrial functions were inseparable from the mitochondria morphology.

The changes of mitochondria morphological were determined by fission and fusion of mitochondria. Therefore, we investigated the fission and fusion of mitochondria. The results illustrated that the mRNA of fission (*drp1*) increased in SN and ACC. Besides, the mitochondrial fusion *mfn1/2* increased and *opa1* was unchanged in SN, while only *mfn2* increased in ACC. *Mfn1* and *opa1* had no significant change or reduced in ACC, respectively. The results suggested that mitochondrial dynamics increased in the SN and shifted toward mitochondrial fission in ACC.

**Figure 6:**
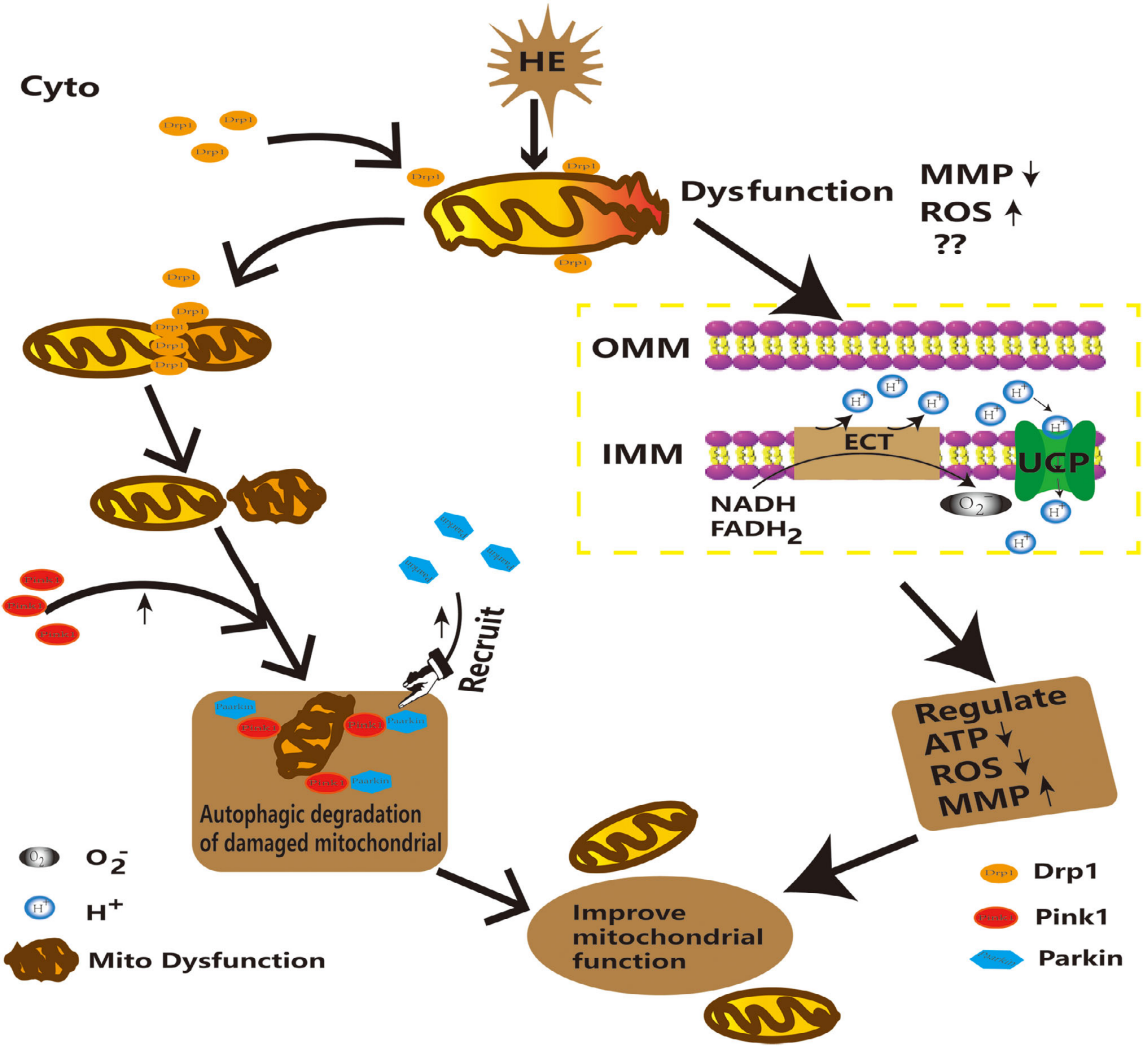
Mitochondrial fission, autophagy and self‐regulation in SN of HE mice. In morphological aspects, the Drp1 protein transfer from the cytoplasm to the mitochondrial outer membrane leads to increase of mitochondrial fission. The autophagy protein Pink1 promotes the recruitment of Parkin resulting in the injured mitochondria to undergo autophagy. In terms of functionality, the uncoupling protein undergoes proton leakage to reduce the amount of oxygen anion (O_2_^−^) generated in the electronic respiratory chain, thereby reducing the production of ROS and the decrease of mitochondrial membrane potential (MMP). Therefore, mitochondria could self‐regulate through fission, autophagy and functional changes to avoid dysfunction and protect the intracellular homeostasis in SN of HE. HE, hepatic encephalopathy; ROS, reactive oxygen species; SN, substantia nigra.

In recent studies revealed that DRP1 could migrate from the cytoplasm to mitochondrion, which promoted mitochondrial fission and fragments (Fig. 6; Ong et al., 2015). MFN1/2 mediated the fusion of the outer mitochondrial membrane (OMM), whereas OPA1 governed the fusion of the inner mitochondrial membrane. However, the MFN2 had been shown to tether the endoplasmic reticulum to mitochondrion which was also related to mitophagy and cell apoptosis (Zhao et al., 2012). So these results suggested that fusion of OMM fusion increased in SN, but that in the inner mitochondrial membrane decreased in ACC. These results demonstrated that in the levels of mRNA the mitochondria had different dynamic and dysfunction in SN from ACC of HE mice. Eventually, damaged mitochondria were removed by autophagy protein.

Mitochondrial autophagy could not only eliminate damaged mitochondria, but also rescue part of the damaged mitochondrial function. There was a close effect on the morphology and function of mitochondria during the development of the disease. The present studies generally considered that Pink1 and Parkin were mainly associated with mitochondrial autophagy (Dagda et al., 2009; Kawajiri et al., 2011; Zhang et al., 2014). Furthermore, a small number of studies suggest that Pink1 and Parkin were also related to mitochondrial fission (Yang et al., 2008; Yu et al., 2011; Rojas‐Charry et al., 2014). Studies had shown that Pink1 and Parkin also participated in the mitochondrial fission process by increasing mitochondrial autophagy (Dagda et al., 2009). However, most of the current researches of Pink1 and Parkin were based on some common neurodegenerative diseases, such as Parkinson disease. We detected the *pink1* and *parkin* in SN and ACC, which were also associated with mitochondrial autophagy in HE mice. The results showed that *pink1* and *parkin* increased in the level of mRNA in SN of HE. However, the *parkin* remarkably reduced in ACC. In addition, oxidative stress had been activated in the brains of human patients and/or in animal models of HE, which damaged the mitochondria physiological function (Mousa et al., 2016; Wu et al., 2016). When mitochondria depolarized, Pink1 (a mitochondrial Ser/Thr kinase) accumulated on the OMM, and recruited Parkin (Fig. 6). The migrations of Pink1 and Parkin to depolarized mitochondria were signs of mitophagy (Kim et al., 2013; Wong and Holzbaur, 2015). Hence, we could boldly suppose that the genes of *pink1* and *parkin* should play an important role in the maintenance of mitochondrial morphology in SN and ACC of HE mice.

ROS was produced in large amounts resulting in mitochondrial dysfunction reported in HE (Dai et al., 2013)**.** Oxidative stress induced mitochondrial fission and caused disturbances in mitochondrial biogenesis that inevitably led to the mitochondrial function damage. Dong et al., (2017) found that abnormal mitochondrial dynamic changes led to mitochondrial regeneration disorders. Disturbances the mitochondrial dynamics in turn resulted in a further increased production of ROS. At present, most studies recognized that UCP2/4 was related to MMP, ROS, Ca^2+^, and ATP production (Kwok et al., 2010; Friederich‐Persson et al., 2012; Wu et al., 2014). Increased ROS and mitochondrial dysfunction would exacerbate the imbalance in the antioxidant system *in vivo*. Some studies had shown that Ucp2 could have protective effects on neurons, reduce oxidative stress and regulate ATP in neurons (Fig. 6; Conti et al., 2005; Islam et al., 2012). Lockwood et al. (1986) found that the metabolic rate of glucose increased in more than 20 brain regions of the rat portacaval shunt model, and the SN and ACC increased 48% and 15%, respectively. While Kosenko found that ATP consumption increased in HE rats (Kosenko et al., 1994). Therefore, we detected the *ucp2* and *ucp4* in SN and ACC of HE mice. The *ucp2* increased in SN and ACC of HE. Interestingly, the *ucp4* was unchanged in SN and reduced in ACC. These results showed that *ucp2* and *ucp4* had significant effects on SN and ACC of HE mice.

## CONCLUSION

In summary, this study provides evidences that mitochondrial dynamics and mitophagy were different in SN and ACC of the HE mice. Manipulation of mitochondrial dynamics and mitophagy signaling could illustrate a new mitochondria strategy to explain the mechanism of HE. Nevertheless, our current results were limited to illustrate an impairment of mitochondrial dynamics, autophagy and function signaling in HE mice. Questions regarding whether and how disrupted mitochondrial regulatory networks influence the pathogenesis of HE are still required further research.
